# Reliability and validity of the Chinese version of the Athletes’ Received Support Questionnaire

**DOI:** 10.3389/fpsyg.2023.1176035

**Published:** 2023-07-27

**Authors:** Ren Yilin, Chu Kequn, Zhu Fengshu

**Affiliations:** ^1^Department of Physical Education, Yangzhou University, Yangzhou, China; ^2^College of Educational Science, Guangxi Science & Technology Normal University, Guangxi, China

**Keywords:** Athletes’ Received Support Questionnaire, reliability, validity, Chinese athletes, Chinese translation

## Abstract

**Objective:**

The aim of this study was to translate the Athletes’ Received Support Questionnaire (ARSQ) for Chinese athletes and examine the reliability and validity of the ARSQ with Chinese subjects.

**Methods:**

In this study, we conducted a forward-backward translation of the ARSQ and used data collected from Chinese athletes to perform exploratory and confirmatory factor analyses. A total of 580 Chinese athletes completed the formal ARSQ for the confirmatory factor analysis (CFA), while 230 athletes participated in the preliminary survey. Of the 580 athletes, 571 provided valid questionnaires for assessing validity and reliability. Additionally, we assessed test–retest reliability using data from 200 participants randomly selected after 1 month. The criterion measurement used in this study was the Social Support Rate Scale (SSRS).

**Results:**

Results of the exploratory factor analysis showed that the Chinese version of the ARSQ with 22 items had four-dimensional structures including emotional support, esteem support, information support, and tangible support. CFA showed that the Chinese version of the ARSQ had adequate structural validity (χ^2^/*df* = 2.315, *CFI* = 0.971, *GFI* = 0.902, *NFI* = 0.959, *AGFI* = 0.878, *RMSEA* = 0.064, *SRMR* = 0.032). Cronbach’s α coefficient, McDonald’s omega coefficient and the test–retest reliability were 0.956, 0.957, and 0.953 for the total scale.

**Conclusion:**

The study provides evidence in support of construct (factorial) validity, convergent validity, internal-consistency and test–retest stability for the use of the ARSQ among Chinese athletes in the Jiangsu and Shandong Provinces, China. However, it is important to note that the conclusion is delimited to the current context, and further studies are needed to verify and promote the applicability of the ARSQ in other regions and sports projects.

## Introduction

Social support is a complex process that involves providing the recipient with a diverse range of resources, such as emotional, informational, and affirmational support in their social relationships, with the aim of helping the recipient achieve personal psychological satisfaction (Langford et al., 1997; Meng et al., 2021). In social psychology, the construct of social support is multifaceted and includes both perceived support and received support. Perceived support refers to the belief that one can obtain necessary help when required, while received support entails the frequency with which an individual obtains support resources within a specific time frame and is typically assessed through retrospective self-reporting (Forenza, 2016). Researchers in sports psychology are particularly interested in exploring the effects of received support on athletes. The nature of the support that athletes receive, whether emotional or informational, and the frequency with which they receive it can have a significant impact on their performance, making the study of social support a complex and multifaceted task (Gottlieb and Bergen, 2010).

In competitive sports, athletes at higher levels are often required to perform under various competitive pressures. Studies have shown that improper handling of athletes’ stress can have many negative effects, such as sports burnout (Wagstaff et al., 2018), overtraining (Meehan et al., 2004) and impaired preparation and performance at sporting competitions (Didymus and Fletcher, 2017). The stress buffering hypothesis posits that received support can act as a buffer to protect athletes from the negative impacts of stressful events, making it an important psychological factor to measure for athletes who experience competitive stress. Specifically, received support can redefine potential threats, enhance personal situational awareness and self-control, promote self-efficacy and better coping ability, and change their emotional or behavioral responses to stress. Received support is a psychological construct (Luo et al., 2017), the frequency with which individuals received support resources at a specific time, and is usually assessed through retrospective self-reports. Specifically, receiving support can redefine the potential threats, enhance personal situational awareness and self-control, promote self-efficacy and better coping ability, and change their emotional or behavioral responses to stress (Freeman and Rees, 2009, 2010; Rees and Freeman, 2009). Received support is widely considered to contribute to the physical and mental health of athletes (Kelly et al., 2011) and is an important resource for athletes to maintain a good competitive state (Cranmer and Sollitto, 2015; Katagami and Tsuchiya, 2017). At the same time, received support has a positive effect on the team cohesion of athletes (Al-yaaribi and Kavussanu, 2017). Empirical evidence also suggests that received support is significantly related to positive athletic performance (Rees and Freeman, 2010), heightened self-confidence during competition (Rees and Freeman, 2007), and effective stress management during competition (Bhadauriya and Tripathi, 2018). In the sports context, research has demonstrated that social support can moderate the effects of competitive stressors on task performance in sport (Rees and Hardy, 2004; Rees and Freeman, 2009). Thus, received support is an essential factor for athletes to cultivate and leverage in order to enhance their competitive readiness and achieve superior athletic outcomes.

Currently, researchers have revised numerous social support measures from various perspectives. However, none of them comprehensively reflect the structural dimensions of support received by athletes, which limits the scope of research on the support received by athletes to some extent. For example, the Social Support Questionnaire (SSQ) (Sarason et al., 1983) measures the number of social support sources and the level of satisfaction with the support received, primarily for social groups in general. The Interpersonal Support Evaluation List (ISEL) (Cohen et al., 1985) assesses college students’ overall competence along four dimensions: authenticity, affiliation, self-evaluation and self-esteem. The Interview Schedule for Social Interaction (ISSI) (Richman et al., 1993) assesses the availability of social support and the suitability of self-perceptions to social relationships in four parts: provider, receiver, interaction, and outcome. It is a measure of the social support process that is developed for clinical use based on the practice model. The Perceived Social Support Scale (PSSS) (Zimet et al., 1988) emphasizes individual self-understanding and self-perception, measuring an individual’s perception of social support from different sources and reflecting the overall level of perceived social support with a total score. Nevertheless, these measures fail to reflect the specific forms of support that athletes require, such as injury management, competition status, technical difficulties, and performance malfunctions, in addition to the support they receive in their daily lives. All of the above scales lack an overall understanding of received support for athletes and do not comprehensively reflect the specific content of athletes’ received support, reducing their application value. Therefore, it is necessary to develop or modify a scale specifically for athletes’ received support.

The Athletes’ Received Support Questionnaire (ARSQ) was developed by Freeman et al. (2014) to address the limitations of previous measures and to propose a four-dimensional theoretical framework for understanding athletes’ received support. The four dimensions include emotional support, esteem support, information support, and tangible support. Emotional support refers to receiving comfort, safety, and personal care, while esteem support refers to obtaining recognition and confidence in one’s abilities from others. Information support refers to receiving helpful advice and guidance, and tangible support refers to obtaining practical and instrumental assistance. The study conducted by Freeman and colleagues provided evidence of the ARSQ’s reliability and cross-cultural generalizability among British athletes.

Receiving support is a crucial aspect of sports, as it can help athletes handle competitive pressures and boost their performance. Researchers in the domains of sports and social psychology have taken a keen interest in the phenomenon of receiving support (Purcell et al., 2019). However, there is currently no Chinese version of the scale to measure received support among Chinese athletes. Therefore, this study aims to revise the ARSQ in Chinese and assess its psychometric properties through exploratory and confirmatory factor analysis (CFA), with the objective of providing a reliable instrument to examine the receipt of support among Chinese athletes. The revision of the scale was performed on a sample of university students in the Chinese cultural context.

## Literature review

Numerous studies have contributed to the understanding of social support and its impact on athletes, particularly in the context of competitive sports. This literature review highlights key studies that have provided insights into the basis and structure of available literature, specifically focusing on the Chinese cultural context. In the Chinese cultural context, Chang et al. (2018) investigated the relationship between social support and mental health in Chinese adolescents. They found that social support, including received support, plays a crucial role in promoting Chinese adolescents’ mental well-being and highlighted the need for further research in the Chinese cultural context. Another study by Zunjia (2016) investigated the role of social support in predicting the physical and mental health outcomes of Chinese athletes. The results indicated that received support was positively associated with athletes’ overall well-being, including lower levels of burnout and stress. The study highlighted the significant contribution of social support to athletes’ holistic health and highlighted the relevance of examining received support specifically in the Chinese cultural context. In the realm of sports performance, Gu and Xue (2022) conducted a study examining the relationship between received support and athletic performance among Chinese athletes. This study investigates the relationships between sports group cohesion, psychological collectivism, mental toughness, and athlete engagement among Chinese team sports athletes. While not solely focused on received support, the study explores the role of cohesion as a form of social support and its association with athlete engagement and performance. Zhang et al. (2021) in their article provides an overview of China’s sport psychology research and practice. It highlights the importance of social support and discusses mental training models proposed by Chinese sport psychologists. While not specifically focused on athletic performance, it offers insights into the field of sport psychology in China. Furthermore, Freeman (2020) provides a comprehensive overview of social support in sport. It discusses the conceptualization and measurement of support, theoretical models such as the main effect model and stress-buffering model, and the effects of social support on outcomes such as burnout, injury prevention, and performance. While not specific to Chinese athletes, it provides insights into the general understanding of social support in sport.

These studies collectively provide a foundation for understanding the importance of social support for Chinese athletes. They highlight the positive impact of received support on athletes’ mental health, physical well-being, athletic performance, and competitive state. However, despite these contributions, there is still a gap in the literature regarding a comprehensive measure of received support specifically tailored to the Chinese cultural context. To address this gap, the study aims to revise the ARSQ in Chinese and evaluate its psychometric properties. By developing a culturally sensitive scale, this research seeks to provide a reliable instrument to assess the receipt of support among Chinese athletes and further contribute to the existing literature on social support in the Chinese cultural context.

## Methods

### Translation

Before revision, we contacted the original author of the ARSQ, obtained permission and translated it using the back-translation method. The original English version of ARSQ was translated into Chinese according to standard guidelines, which are widely accepted to successfully translate measures in cross-cultural research (Behr, 2017). First, two English-Chinese bilingual researchers were asked to independently translate the English version of the scale into Chinese to develop a preliminary Chinese version of the scale. Subsequently, the translated version was sent to two Chinese exercise psychologists with good bilingual skills (Chinese and English) who reviewed and provided feedback on this version. Based on their feedback, this newly translated version was revised and then sent to two individuals fluent in English and Chinese and invited to independently perform back-translation. Then, two native English speakers (bilingual in English and Chinese) were invited to translate Chinese into English. Before all translators and researchers came to an agreement, any differences between the original version and the back translated version had been discussed. Then, using the original version, the preliminary Chinese version and the back translated English version of the scale, the Chinese version of the ARSQ for Chinese athletes was formed by comparing the items one by one and considering the words used. The final items and scoring method of the scale were considered consistent with the original questionnaire.

### Participants

Our research aimed to recruit Chinese athletes from three sports colleges located in Jiangsu and Shandong provinces, China, using a multistage cluster sampling method. First, we randomly selected the three sports colleges from a list of all sports colleges in the two provinces. Then, classes were randomly chosen from each of the selected colleges, and all athletes who were enrolled in the selected classes were invited to partake in the study. The study participants engaged in various forms of exercise (e.g., sprint, middle-distance running, throwing, soccer, volleyball, basketball, gymnastics, judo). Demographic data on age, gender, self-reported height and weight, and training years were collected (Tables 1, 2). Data were collected through an online cross-sectional survey in November 2022. Initially, the Dean of Student Affairs at each college contacted eligible participants and shared a brief invitation letter along with a link to an online questionnaire. Subsequently, all participants received a short invitation message and link to the online survey via WeChat (a multifunctional mobile application). The survey consisted of two parts: the first part included an introduction to the study and an informed consent form, while the second part included the Chinese version of the ARSQ. The study commenced after the participants completed the informed consent form and agreed to participate. Only those participants who completed the informed consent form and agreed to participate were included in the sample. The sample size satisfied the requirements for factor analysis and other psychological measurement evaluations (Floyd and Widaman, 1995). The sample was divided into prediction samples, formal samples, and test–retest samples.

**Table 1:** Participant characteristics for prediction sample (sample 1, male 122 female 97).

Gender	Item	Number/person	Age/year	Height/cm	Weight/kg	Training years/a
Male	Sprint	18	21.4 ± 1.65	178.8 ± 3.42	66.8 ± 3.43	7.2 ± 2.54
	Middle-distance running	22	20.6 ± 2.13	175.2 ± 4.43	63.2 ± 3.58	8.2 ± 1.68
	Throwing	9	22.7 ± 1.69	181.2 ± 4.17	82.7 ± 6.96	7.5 ± 2.18
	Soccer	16	20.3 ± 1.13	182.2 ± 4.27	77.4 ± 5.24	6.3 ± 2.12
	Volleyball	15	20.2 ± 3.67	186.2 ± 5.36	78.6 ± 5.75	8.2 ± 2.43
	Basketball	21	21.8 ± 3.32	186.9 ± 4.37	79.5 ± 5.25	7.9 ± 3.22
	Gymnastics	13	19.8 ± 2.54	165.3 ± 3.52	55.2 ± 3.58	8.5 ± 2.75
	Judo	8	22.1 ± 2.46	175.6 ± 3.24	80.8 ± 6.75	7.3 ± 2.23
Female	Sprint	13	21.6 ± 1.21	172.6 ± 3.55	61.2 ± 4.23	6.8 ± 2.34
	Middle-distance running	15	22.3 ± 2.42	168.4 ± 4.12	58.4 ± 3.66	7.7 ± 2.82
	Throwing	8	22.5 ± 2.61	169.8 ± 4.18	73.4 ± 5.17	7.3 ± 2.32
	Soccer	12	21.8 ± 2.78	174.2 ± 4.54	67.8 ± 5.14	6.5 ± 2.16
	Volleyball	14	21.1 ± 3.32	182.9 ± 4.23	67.2 ± 4.76	6.8 ± 1.88
	Basketball	16	21.4 ± 3.26	182.6 ± 5.66	68.7 ± 5.27	6.7 ± 1.87
	Gymnastics	14	19.2 ± 2.14	161.3 ± 3.32	46.2 ± 3.37	8.7 ± 1.67
	Judo	5	22.3 ± 2.32	170.6 ± 4.27	71.4 ± 4.84	5.6 ± 2.34

**Table 2:** Participant characteristics for formal sample (sample 2, male 359 female 212).

Gender	Item	Number/person	Age/year	Height/cm	Weight/kg	Training years/a
Male	Sprint	43	21.2 ± 1.43	178.8 ± 2.74	65.7 ± 3.54	7.6 ± 2.14
	Middle-distance running	41	20.3 ± 3.32	175.1 ± 4.31	62.8 ± 3.64	8.4 ± 1.58
	Throwing	29	21.7 ± 2.34	182.3 ± 3.87	82.9 ± 7.17	7.4 ± 2.68
	Soccer	55	21.3 ± 2.73	183.2 ± 4.34	76.6 ± 5.45	6.2 ± 2.37
	Volleyball	63	20.2 ± 3.56	185.2 ± 5.67	77.6 ± 5.71	7.9 ± 3.43
	Basketball	64	20.8 ± 3.47	186.9 ± 5.49	80.2 ± 5.63	7.8 ± 3.36
	Gymnastics	38	19.6 ± 2.34	164.8 ± 3.76	54.6 ± 3.48	8.8 ± 2.64
	Judo	26	22.3 ± 2.47	174.5 ± 3.52	81.7 ± 6.81	7.1 ± 2.13
Female	Sprint	18	20.4 ± 1.37	171.2 ± 3.41	60.8 ± 4.11	6.4 ± 2.27
	Middle-distance running	26	21.7 ± 2.14	167.4 ± 4.15	57.4 ± 3.76	7.3 ± 2.84
	Throwing	15	21.9 ± 3.08	168.7 ± 4.23	72.6 ± 5.27	7.1 ± 2.58
	Soccer	28	20.6 ± 3.56	174.7 ± 4.57	65.3 ± 5.72	6.2 ± 2.46
	Volleyball	37	20.8 ± 3.21	181.6 ± 4.28	66.9 ± 4.35	6.5 ± 1.94
	Basketball	36	20.5 ± 3.41	182.7 ± 5.72	67.6 ± 5.26	6.3 ± 1.97
	Gymnastics	44	18.3 ± 2.12	160.4 ± 3.48	44.7 ± 3.38	7.2 ± 1.46
	Judo	8	21.1 ± 3.35	169.8 ± 4.37	70.3 ± 4.47	4.9 ± 2.65

Sample 1 (prediction sample, used for item analysis and exploratory factor analysis): 230 questionnaires were randomly distributed to sport colleges in Jiangsu and Shandong provinces, China, including both students and faculty members. A total of 219 valid responses were received, resulting in a response rate of 95%. The sample consisted of 122 males and 97 females, with an age range of 17–25 years. All participants had obtained at least a bachelor’s degree (Table 1).

Sample 2 (formal samples, used for CFA and reliability assessment): A total of 580 Chinese athletes (all of them are student athletes) were selected from three Sports colleges in Jiangsu and Shandong provinces using a convenient sampling method cluster random sampling method. Questionnaires were distributed, and 571 valid questionnaires were collected, yielding a response rate of 98%. The sample consisted of 359 males and 212 females, aged between 16 and 26 years, all of whom had attained an undergraduate or higher education (Table 2). One month later, 150 participants were randomly selected from Sample 2 for retesting to examine the test–retest reliability.

### Measures

#### The Athletes’ Received Support Questionnaire

The instrument employed in this study was originally developed by Freeman et al. (2014) and used the Chinese version of the ARSQ to measure the extent to which athletes receive social support. The ARSQ includes four factors and 22 items in total, specifically: emotional support is measured by 5 items, esteem support is measured by 5 items, information support is measured by 6 items, tangible support is measured by 6 items. The scale is scored on a 5-point scale (1 not even once, 2 once or twice, 3 three or four times, 4 five or six times, 5 seven or more times), with higher scores on each dimension representing the more support received by the athlete.

#### Calibration tool

The Social Support Rate Scale (SSRS) (Shuiyuan, 1994) was used to construct the scaling framework by using the social support theory of subjective support (the individual can experience or emotional support) and objective support (the substantive support received by the individual), combined with the support utilization (reflecting the individual’s active use of various social support). The scale consists of 10 items, including objective support (3 items), subjective support (4 items), and use of social support (3 items). The scale scoring method is that the total score is the sum of the scores of 10 items; the objective support score was the sum of the scores of 2, 6, and 7. The subjective support score was the sum of scores 1, 3, 4, and 5. Support utilization was computed as the sum of ratings 8, 9, and 10. In Articles 1–4 and 8–10, the scoring approach involved choosing one item per category, where items 1–4 were assigned scores of 1–4 points, respectively. Article 5’s score was computed by totaling A, B, C, and D, with each item receiving a score from zero to full support (1–4 points). Articles 6 and 7 were scored as 0 if the answer was “no source,” while multiple sources were tallied if the response was “the following source.” The degree of social support increased with higher scores on the total scale and subscales. The SSRS total scale in this study had good reliability and validity, with Cronbach’s α coefficients ranging from 0.83 to 0.90. The objective support had a coefficient of 0.87, the subjective support had a coefficient of 0.83, and the utilization of support had a coefficient of 0.90.

### Data analysis

This study utilized SPSS 27.0 to conduct descriptive statistics, which provided important information about central tendencies and detailed insights into the present data, including mean score, standard deviation, kurtosis, and skewness. Kurtosis and skewness values that ranged between −1.5 and +1.5 were defined as a normal distribution (Byrne and Campbell, 1999). Additionally, SPSS 27.0 was employed for exploratory factor analysis (EFA) to evaluate the internal consistency of the scale, measured through both Cronbach’s alpha coefficient and McDonald’s omega coefficient, as well as construct (factorial) validity, convergent validity, internal-consistency, and test–retest stability. Second, Amos 24.0 was used for CFA. To determine the stability of the four-factor model, we also tested uni-variate model, bi-factor models, and five-factor model for comparison. The determination of model fit is based on several criteria: Chi-square, standardized root means square residual (SRMR), the root mean squared error of approximation (RMSEA), comparative fit index (CFI), goodness-of-fit index (GFI), normed fit index (NFI) as well as the adjusted goodness-of-fit index (AGFI).

## Results

### Data normality

With respect to the ARSQ, data on means, standard deviations, kurtosis and skewness are presented in Table 3. The value of kurtosis and skewness of each item in whole sample ranged from −1.224 to 0.717, indicating all 22 items are normally distributed (Muthén and Kaplan, 1985). Of 571 participants, there were 359 male and 212 female athletes. Given the normal distribution of the data, the researchers used Structural Equation Modeling (SEM) as the estimation method in their latent variable model. It is important to consider the normality of the data when selecting the appropriate statistical analysis for the research question, as deviations from normality can affect the accuracy of the results.

**Table 3:** Descriptive analysis of translated items of ARSQ.

Factor	Item	All (*n* = 571)				Male (*n* = 359)				Female (*n* = 212)			
		Mean	SD	Skewness	Kurtosis	Mean	SD	Skewness	Kurtosis	Mean	SD	Skewness	Kurtosis
1. Emotional support	1	2.36	1.042	0.518	0.230	2.35	0.99	0.576	0.604	2.37	1.13	0.440	−0.233
	2	2.78	1.073	0.437	0.089	2.84	1.04	0.514	0.202	2.69	1.12	0.372	−0.079
	3	3.01	1.077	0.480	−0.160	3.10	1.05	0.517	−0.201	2.85	1.10	0.486	−0.087
	4	2.82	1.159	0.438	−0.291	2.85	1.15	0.493	−0.235	2.76	1.18	0.365	−0.383
	5	2.76	1.041	0.462	0.247	2.78	0.98	0.519	0.553	2.71	1.14	0.428	−0.135
2. Esteem support	6	2.96	1.400	0.311	−1.224	3.06	1.37	0.252	−1.265	2.79	1.43	0.440	−1.122
	7	2.83	1.372	0.466	−1.037	2.84	1.35	0.530	−0.949	2.83	1.42	0.375	−1.168
	8	2.83	1.389	0.424	−1.078	2.87	1.36	0.412	−1.045	2.76	1.43	0.460	−1.121
	9	2.76	1.406	0.498	−1.025	2.80	1.37	0.516	−0.953	2.71	1.47	0.490	−1.130
	10	2.75	1.412	0.484	−1.044	2.79	1.39	0.496	−1.012	2.69	1.45	0.482	−1.094
3. Informational support	11	2.67	1.252	0.228	−1.074	2.60	1.21	0.290	−1.011	2.77	1.32	0.112	−1.166
	12	2.74	1.198	0.221	−0.976	2.73	1.14	0.242	−0.889	2.76	1.29	0.188	−1.127
	13	2.76	1.200	0.195	−0.985	2.73	1.15	0.219	−0.935	2.82	1.27	0.141	−1.081
	14	2.73	1.206	0.275	−0.988	2.70	1.14	0.292	−0.896	2.78	1.31	0.226	−1.161
	15	2.68	1.189	0.282	−0.962	2.67	1.15	0.310	−0.937	2.71	1.25	0.236	−1.017
	16	2.73	1.196	0.241	−1.010	2.69	1.15	0.264	−0.925	2.81	1.27	0.179	−1.157
4. Tangible support	17	2.06	1.132	1.071	0.580	1.97	1.05	1.101	0.894	2.21	1.25	0.946	0.010
	18	2.08	1.158	1.081	0.532	2.00	1.09	1.169	0.959	2.23	1.25	0.921	−0.040
	19	2.10	1.157	1.072	0.530	2.03	1.09	1.137	0.895	2.23	1.26	0.939	0.001
	20	2.05	1.137	1.120	0.682	1.96	1.04	1.192	1.164	2.21	1.27	0.943	−0.028
	21	2.07	1.132	1.111	0.685	1.98	1.03	1.181	1.209	2.21	1.27	0.933	−0.069
	22	2.04	1.138	1.140	0.717	1.95	1.04	1.203	1.186	2.20	1.27	0.971	0.003
ARSQ Total	Total	2.66	0.553	0.561	−0.418	2.60	0.52	0.860	0.910	2.65	1.16	0.270	−0.580

#### Item analysis

Analyzed data from Predictor Sample 1 underwent item analysis. Correlation tests were executed on all 22 items, and the results indicated that the total correlation coefficient of all 22 items was positively correlated and exceeded 0.40 (see Table 4). Furthermore, the correlation coefficient of each item with the overall scale score ranged from 0.428 to 0.759 (all *p* < 0.001). Therefore, all 22 items met the retention criteria, and no items were excluded from the item analysis. The present study employed the polychoric correlation coefficient to calculate the correlations between the 22 items in Predictor Sample 1. This correlation coefficient was deemed suitable for ordinal data and was preferred over other widely used coefficients, such as Pearson’s correlation coefficient, as it yields a more precise estimate of the actual correlation.

**Table 4:** The ARSQ correlation matrix.

Correlation matrix	a1	a2	a3	a4	a5	a6	a7	a8	a9	a10	a11	a12	a13	a14	a15	a16	a17	a18	a19	a20	a21	a22
a1	1																					
a2	0.497^**^	1																				
a3	0.345^**^	0.613^**^	1																			
a4	0.403^**^	0.594^**^	0.683^**^	1																		
a5	0.514^**^	0.608^**^	0.657^**^	0.760^**^	1																	
a6	0.278^**^	0.337^**^	0.390^**^	0.437^**^	0.491^**^	1																
a7	0.218^**^	0.298^**^	0.315^**^	0.373^**^	0.392^**^	0.825^**^	1															
a8	0.222^**^	0.315^**^	0.329^**^	0.395^**^	0.411^**^	0.866^**^	0.860^**^	1														
a9	0.236^**^	0.336^**^	0.309^**^	0.380^**^	0.402^**^	0.862^**^	0.832^**^	0.884^**^	1													
a10	0.241^**^	0.319^**^	0.305^**^	0.369^**^	0.391^**^	0.854^**^	0.855^**^	0.861^**^	0.917^**^	1												
a11	0.232^**^	0.332^**^	0.289^**^	0.342^**^	0.366^**^	0.427^**^	0.450^**^	0.421^**^	0.463^**^	0.488^**^	1											
a12	0.231^**^	0.363^**^	0.340^**^	0.394^**^	0.424^**^	0.485^**^	0.475^**^	0.449^**^	0.500^**^	0.511^**^	0.855^**^	1										
a13	0.208^**^	0.355^**^	0.358^**^	0.392^**^	0.420^**^	0.454^**^	0.461^**^	0.435^**^	0.477^**^	0.491^**^	0.848^**^	0.910^**^	1									
a14	0.247^**^	0.349^**^	0.324^**^	0.387^**^	0.404^**^	0.467^**^	0.456^**^	0.450^**^	0.480^**^	0.504^**^	0.869^**^	0.893^**^	0.897^**^	1								
a15	0.233^**^	0.332^**^	0.299^**^	0.359^**^	0.375^**^	0.448^**^	0.449^**^	0.439^**^	0.464^**^	0.475^**^	0.862^**^	0.856^**^	0.872^**^	0.897^**^	1							
a16	0.229^**^	0.323^**^	0.313^**^	0.377^**^	0.372^**^	0.445^**^	0.442^**^	0.445^**^	0.481^**^	0.477^**^	0.848^**^	0.847^**^	0.874^**^	0.872^**^	0.889^**^	1						
a17	0.281^**^	0.377^**^	0.335^**^	0.391^**^	0.434^**^	0.429^**^	0.424^**^	0.453^**^	0.462^**^	0.457^**^	0.510^**^	0.485^**^	0.500^**^	0.493^**^	0.544^**^	0.542^**^	1					
a18	0.289^**^	0.385^**^	0.330^**^	0.382^**^	0.401^**^	0.414^**^	0.420^**^	0.461^**^	0.462^**^	0.453^**^	0.501^**^	0.485^**^	0.498^**^	0.490^**^	0.529^**^	0.523^**^	0.892^**^	1				
a19	0.286^**^	0.390^**^	0.335^**^	0.375^**^	0.409^**^	0.419^**^	0.429^**^	0.453^**^	0.457^**^	0.451^**^	0.497^**^	0.481^**^	0.504^**^	0.500^**^	0.529^**^	0.515^**^	0.872^**^	0.910^**^	1			
a20	0.295^**^	0.383^**^	0.324^**^	0.370^**^	0.408^**^	0.403^**^	0.407^**^	0.425^**^	0.444^**^	0.441^**^	0.504^**^	0.475^**^	0.502^**^	0.498^**^	0.545^**^	0.517^**^	0.880^**^	0.903^**^	0.898^**^	1		
a21	0.284^**^	0.385^**^	0.306^**^	0.365^**^	0.413^**^	0.416^**^	0.429^**^	0.435^**^	0.455^**^	0.452^**^	0.488^**^	0.502^**^	0.499^**^	0.494^**^	0.517^**^	0.523^**^	0.869^**^	0.872^**^	0.867^**^	0.911^**^	1	
a22	0.254^**^	0.378^**^	0.309^**^	0.344^**^	0.377^**^	0.415^**^	0.422^**^	0.437^**^	0.457^**^	0.442^**^	0.464^**^	0.462^**^	0.457^**^	0.466^**^	0.491^**^	0.495^**^	0.866^**^	0.859^**^	0.856^**^	0.896^**^	0.901^**^	1

^**^Indicates that there is a correlation between the product differences of individual topics. *p* < 0.01 (two-tailed), the correlation is significant.

#### Exploratory factor analysis

**Figure 1:**
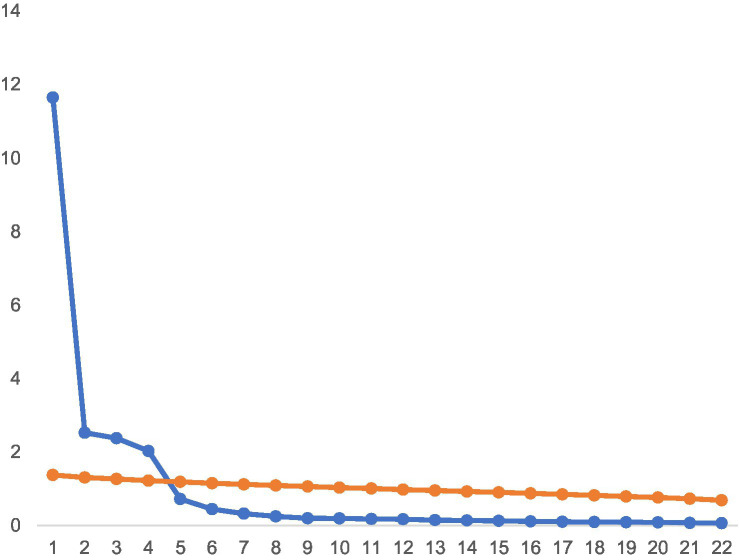
Scree plot and parallel analysis.

The present study conducted factor analysis on 22 items to uncover underlying dimensions. Principal component analysis (PCA) was chosen for the purpose of capture the maximum amount of variation in the data with the minimum number of components. Notably, a Kaiser-Meyer-Olkin (KMO) measure of 0.949 was obtained using PCA and Promax diagonal rotation, surpassing the minimum threshold of 0.5, which supports the suitability of factor analysis for the current dataset. Further, results of the Bartlett’s sphericity test was statistically significant (*χ*^2^ = 16352.797, *p* < 0.01), signifying that factor analysis was deemed appropriate. In combination with the scree plot and Parallel analysis (Figure 1), the red line is the factor extracted for the parallel analysis, and the blue line is the factor extracted for your real data. The points on the blue line are above the red line, suggesting the factors that should be extracted. Based on the results of the parallel analysis, we can conclude that it is better to extract 4 factors. The number of factors according to the original MAP Test (Velicer, 1976) is 4 and the revised MAP Test (Velicer et al., 2000) is 4. Hence, four main factors (Table 5) were obtained, including Factor 1, Tangible Support; Factor 2, Information Support; Factor 3, Esteem Support; Factor 4, Emotional Support.

**Table 5:** Rotated component matrix.^a^

Item	Component				Communality
	Factor 1	Factor 2	Factor 3	Factor 4	
a1				0.631	0.435
a2				0.768	0.671
a3				0.794	0.688
a4				0.802	0.742
a5				0.813	0.780
a6			0.861		0.882
a7			0.864		0.860
a8			0.887		0.902
a9			0.881		0.909
a10			0.878		0.907
a11		0.862			0.867
a12		0.869			0.896
a13		0.881			0.909
a14		0.886			0.917
a15		0.871			0.900
a16		0.861			0.883
a17	0.861				0.889
a18	0.879				0.909
a19	0.87				0.897
a20	0.894				0.928
a21	0.878				0.904
a22	0.882				0.892
Total	11.647	2.522	2.372	2.025	
% of Variance	53.939	11.462	10.781	9.206	
Cumulative %	52.939	64.401	75.182	84.388	

Extraction method: principal component analysis. Rotation method: varimax with Kaiser normalization.

^a^Rotation converged in 6 iterations.

#### Confirmatory factor analysis

**Figure 2:**
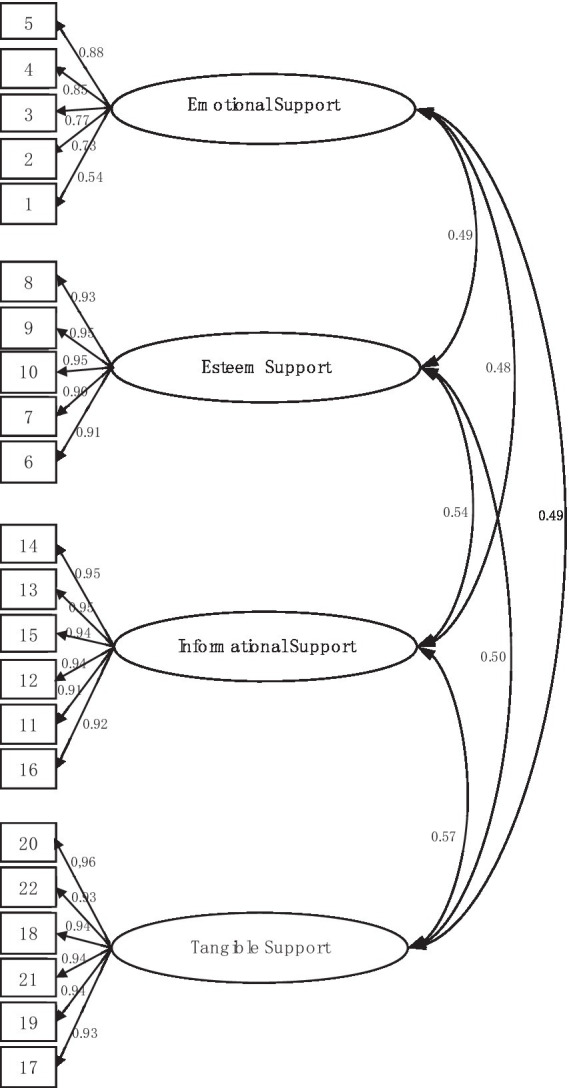
Statistical diagram of the structural model of the ARSQ (four-factor model).

A four-factor model was compared with a uni-variate model, bi-factor model and five-factor model, respectively. Results indicate that the four factor model shows an adequate fit (*χ*^2^*/df* = 2.135, *p* < 0.01, *CFI* = 0.971, *GFI* = 0.902, *NFI* = 0.959, *AGFI* = 0.878, *RMSEA* = 0.064, *SRMR* = 0.032), whereas the uni-factor structure (*χ*^2^/*df* = 15.674, *CFI* = 0.444, *GFI* = 0.297, *NFI* = 0.430, *AGFI* = 0.149, *RMSEA* = 0.272, *SRMR* = 0.301) and bi-factor structure (*χ*^2^/*df* = 9.027, *CFI* = 0.691, *GFI* = 0.475, *NFI* = 0.667, *AGFI* = 0.361, *RMSEA* = 0.203, *SRMR* = 0.186) are insufficient. To explore the possibility of simplifying the four-factor model, a higher-order factor (five-factor structure) was also tested. The results showed that the five-factor structure was reasonable, but slightly worse than the four-factor structure (*χ*^2^/*df* = 4.826, *CFI* = 0.862, *GFI* = 0.732, *NFI* = 0.833, *AGFI* = 0.660, *RMSEA* = 0.139, *SRMR* = 0.179). Results of the four different models are presented in Table 6 and the structure is presented in Figure 2.

**Table 6:** Goodness-of-fit indices from CFA in four models (*n* = 571).

	χ^2^/df	CFI	GFI	NFI	AGFI	RMSEA	SRMR
Uni-variate model	15.674	0.444	0.297	0.430	0.149	0.272	0.301
Bi-factor model	9.027	0.691	0.475	0.667	0.361	0.203	0.186
Five-factor model	4.826	0.862	0.732	0.833	0.660	0.139	0.179
Four-factor model	2.315	0.971	0.902	0.959	0.878	0.064	0.032

χ^2^/df, Chi-Square Test of Model Fit/degrees of freedom; CFI, Comparative Fit Index; GFI, Goodness-of-Fit index; NFI, Normed Fit index; AGFI, Adjusted Goodness-of-Fit index; RMSEA, Root Mean Square Error of Approximation; SRMR, Standardized Root Mean Square Residual.

#### Criterion-related validity

In this study, the relationship between the ARSQ and the Student Social Support Scale (SSRS) were used to test construct validity. As presented in Table 7, the ARSQ total score was positively related to SSRS (*r* = 0.43, *p* < 0.01). Furthermore, the correlation coefficients between the scores of each dimension of the ARSQ and the total score of the SSRS were all statistically significant, ranging from 0.32 to 0.45 (all *p* < 0.01).

**Table 7:** Analysis of correlation between ARSQ and SSRS.

	ARSQ	SSRS
ARSQ	1	
1. Emotional support		0.32^**^
2. Esteem support		0.36^**^
3. Informational support		0.45^**^
4. Tangible support		0.41^**^
SSRS	0.43^**^	1

ARSQ, Athlete Received Support Questionnaire; SSRS, Student Social Support Scale. ^**^p < 0.01.

#### Reliability evaluation

The value of Cronbach’s α and McDonald’s ω is usually used to assess internal consistency reliability. Generally, the Cronbach’s α and McDonald’s ω of greater than 0.7 indicates that the internal consistency reliability of the scale is adequate (Floyd and Widaman, 1995). Results indicated that the value of Cronbach’s α and McDonald’s ω involved emotional support (0.868, 0.873, good), esteem support (0.969, 0.969, excellent), information support (0.976, 0.976, excellent), and tangible support (0.978, 0.979, excellent) (see Table 8). Moreover, all items in the scale were positively correlated (*r* = 0.208–0.917, *p* < 0.01). Furthermore, all items in the scale were positively correlated with total score (*r* = 0.254–0.901, *p* < 0.01) (Table 3).

**Table 8:** Internal consistency reliability and retest reliability of the Chinese version of the ARSQ after 4 weeks.

Item	Cronbach’s α (*n* = 571)	McDonald’s ω (*n* = 571)	Test–retest reliability (*n* = 200)
ARSQ	0.956	0.957	0.953
Emotional support	0.868	0.873	0.833
Esteem support	0.969	0.969	0.972
Information support	0.976	0.976	0.972
Tangible support	0.978	0.979	0.952

After a four-week interval, 200 participants were randomly selected from samples 2 to assess the test–retest stability of the ARSQ. The retest data revealed correlation coefficients ranging from 0.833 to 0.972 between the total scale score and each dimension of the ARSQ. The test–retest reliability of the ARSQ total scale was found to be 0.953, indicating high stability over time. The four subscales also showed high test–retest reliabilities, with values of 0.833, 0.972, 0.972, and 0.952 (see Table 3).

## Discussion

Received support has been shown to have many positive effects on athletes and coaches, but there has been a paucity of evidence to support a comprehensive measurement (Katagami and Tsuchiya, 2016; Simons and Bird, 2022). This study aimed to investigate the applicability of the Chinese version of the ARSQ in the Chinese athlete population by collecting data from athletes in universities located in the Jiangsu and Shandong provinces. To achieve this goal, the Chinese version of the ARSQ underwent revisions through the translation of the athlete acceptance support scale, exploratory factor analysis, reliability and validity tests, and CFA. This study provides support for the construct and criterion-related validity and internal consistency and test–retest reliability of the ARSQ in a student athlete population in the Jiangsu and Shandong provinces. Additionally, the exploratory factor analysis and project analysis indicated that the commonality of 22 items was statistically significant, allowing for the retention of these items from the original scale. Furthermore, the CFA demonstrated a stable four-factor structure: emotional support, esteem support, information support, and tangible support, which was consistent with the original scale. The internal consistency coefficients of the overall scale and the four dimensions were all above 0.868, indicating high reliability. These results reflect the effectiveness of the ARSQ in measuring the received support of Chinese athletes.

This study also found that the average score of the ARSQ and its four subscales showed a significant positive correlation with the SSRS score. This result was in line with previous studies that have demonstrated the noteworthy predictive effect of receiving support on an athlete’s competitive status and athletic performance. Studies have shown that a higher positive level of support leads to better competitive status and athletic performance during competitions. The correlation of the scores of the ARSQ scale and its subscales with the SSRS scores in this study implied that the Chinese version of the revised ARSQ scale has higher criterion-related validity. The result was in line with previous studies that have emphasized the importance of social support for athletes and its impact on various aspects of their performance. For example, some studies have reported that athletes who received high levels of social support from their coaches and teammates trend to demonstrate higher levels of confidence, self-efficacy, and motivation, ultimately leading to better performance outcomes (Davis et al., 2018; McLean and Penco, 2020). Similarly, a study by Freeman et al. (2014) found that social support from coaches and teammates had a positive effect on athletes’ mental toughness and ability to cope with stress during competitions. The correlation of the ARSQ scale and its subscales with the SSRS scores in this study also suggests that the Chinese version of the revised ARSQ scale is a valid and reliable instrument for measuring social support in Chinese athletes. This finding is consistent with previous studies that have validated the ARSQ scale in other cultural contexts, including the United States (Freeman et al., 2014). Overall, these results underscore the importance of receive support fundamental determinant of athletes’ well-being and performance enhancement in competitive sports. Additionally, this investigation also subjected 200 individuals in the formal measured samples to a retest. The retest data demonstrated that the retest reliability of the ARSQ total scale was 0.953, and the retest reliabilities (*r*) of the four subscales were 0.833, 0.972, 0.972, and 0.952. These findings indicate that the scale has good stability.

This study has important theoretical and practical implications. The theoretical implications include advancing the understanding of received support by validating the Chinese version of the ARSQ, thereby contributing to the knowledge on received support in the Chinese athlete population. The identification of emotional support, esteem support, information support, and tangible support as stable dimensions within the ARSQ demonstrates cross-cultural consistency in the conceptualization of received support. Moreover, the positive correlation between the ARSQ scores and the SSRS scores reinforces the theoretical proposition that creating a supportive environment within sports organizations is crucial for athletes’ well-being and performance outcomes. From the practical perspective, the validated ARSQ serves as a reliable measurement tool for assessing social support among Chinese athletes. This enables researchers and practitioners to evaluate the level of support received, identify areas for improvement in support systems, and assess the effectiveness of support interventions. Additionally, the study highlights the importance of implementing support programs that enhance athletes’ access to emotional, esteem, information, and tangible support. Such programs can positively influence athletes’ psychological well-being, motivation, self-efficacy, and ultimately their performance outcomes. Moreover, prioritizing social support in athletes’ lives through interventions and support programs can contribute to their overall well-being, confidence, and resilience. Lastly, the validation of the ARSQ in the Chinese cultural context emphasizes the need for culturally sensitive approaches when designing athlete support programs.

Overall, this study provides theoretical advancements by expanding the understanding of received support in the Chinese athlete population and has practical implications for the measurement of social support, the design of athlete support programs, and the recognition of cultural context in support interventions. These findings contribute to the field of sports psychology and support the development of effective support systems for athletes.

## Strengths and limitations

The revised Chinese version of the four-dimensional ARSQ demonstrated to have high levels of reliability and validity, making it an effective measurement tool for evaluating social support and its impact on athletes in the Chinese context. Nevertheless, some limitations have to be acknowledged. First, this study is limited by its sample composition, which was limited to college athletes from Jiangsu and Shandong provinces in China. Therefore, caution should be exercised in generalizing the results to other populations, and further research is needed to examine the applicability and generalizability of the scale in different cultural and sample contexts. Thus, this study does not account for the potential influence of social support on athletes, and future research should explore the relationship between social support and individual perceptions of support received. Thirdly, this study focused on the support that athletes receive in non-competitive contexts, and did not examine the unique nature and effects of support during actual competitions. Therefore, future research should take into account the impact of competition-specific support on athletes, in order to gain a more comprehensive understanding of the role of support in athletes’ performance and well-being.

## Conclusion

We were able to demonstrate a concise and theory-based self-report assessment tool, aiming to measure the various types of support that athletes receive during both training and competition, including emotional, esteem, informational, and tangible support. It measures the different types of support athletes receive during training and competition. The study shows that the revised Chinese version of the ARSQ has good psychometric properties, indicating its usefulness as a tool for measuring athletes’ support levels in the Chinese context. However, the study’s scope is limited to college athletes from Jiangsu and Shandong provinces in China, and caution should be exercised in generalizing the results to other populations. The ARSQ serves as a practical tool for researchers and practitioners to assess and enhance athletes’ support systems, ultimately promoting their well-being and optimizing their athletic performance. Further research is needed to examine the cross-cultural validity and applicability of the scale in different contexts and to explore the unique nature and effects of support during actual competitions.

## Data availability statement

The original contributions presented in the study are included in the article/supplementary material, further inquiries can be directed to the corresponding author.

## Ethics statement

The study was approved by the Ethics Committee of Yangzhou University on November 1, 2022. The patients/participants provided their written informed consent to participate in this study.

## Author contributions

RY was responsible for project implementation, experiment design, and thesis writing. CK was responsible for data analysis and statistical results. ZF was responsible for project supervision and thesis revision. All authors contributed to the article and approved the submitted version.

## Funding

This research was supported by the National Social Science Fund Project [Title: Construction and Application of Targeted Movement Intervention Model for Adolescent Aggressive Behavior (20BTY118)].

## Conflict of interest

The authors declare that the research was conducted in the absence of any commercial or financial relationships that could be construed as a potential conflict of interest.

## Publisher’s note

All claims expressed in this article are solely those of the authors and do not necessarily represent those of their affiliated organizations, or those of the publisher, the editors and the reviewers. Any product that may be evaluated in this article, or claim that may be made by its manufacturer, is not guaranteed or endorsed by the publisher.

## Figures and Tables

**Table:** 

类型	项目编号	项目描述
情感支持(Emotion support)	1	在过去的一周中，有人会为您加油多少次 (cheer you up)
	2	在过去的一周中，有人多久会倾听您 (listen to you)
	3	在过去的一周中，某人多久对您表示关注 (show concern for you)
	4	在过去的一周中，某人多久让您感到他们会一直在您身边(make you feel that they would always be there for you)
	5	在过去的一周中，某人多久安慰一次您 (comfort you)
尊重支持(Esteem support)	6	在过去的一周中，有人多久鼓励您一次 (encourage you)
	7	在过去的一周中，某人多久强调一次您的能力 (emphasize your abilities)
	8	在过去的一周中，有人告诉您多少次您可以做到 (tell you, you can do it)
	9	在过去的一周中，某人多久增强一次积极的一面 (reinforce the positives)
	10	在过去的一周中，某人多久增强一次您的信心 (boost your confidence)
信息支持(Information support)	11	在过去的一周中，有人多久给您一些有关在竞争情况下的表现的建议(give you advice about performing in competitive situations)
	12	在过去的一周中，有人多久给您一次战术建议 (give you tactical advice)
	13	在过去的一周中，某人多久向您提供一次想法并提出建议(offer you ideas and suggest actions)
	14	在过去的一周中，有人多久帮助您看一次事情 (help you put things in perspective)
	15	在过去的一周中，某人多久帮助您决定要做什么 (help you decide what to do)
	16	在过去的一周中，有人多久给您一些有关该做什么的建议 (give you advice about what to do)
确认支持(Tangible support)	17	在过去的一周中，某人多久帮助您计划一次培训 (help plan your training)
	18	在过去的一周中，某人多久协助运送到训练和比赛/比赛(help with transport to training and competition/matches)
	19	在过去的一周中，某人多久在一次培训和比赛/比赛中为您做事(do things for you at training and competitions/matches)
	20	在过去的一周中，某人多久帮助安排一次培训课程 (help set sessions in training)
	21	在过去的一周中，某人多久帮助您完成一项任务 (help you with tasks)
	22	在过去的一周中，有人多久帮助您管理一次培训课程(help manage your training sessions)

## Appendix 1

The Chinese version and the original English version of the Athletes’ Received Support Questionnaire for athletes.

评分:1一次也没有; 2一次或两次; 3三次或四次; 4五次或六次; 5七次或更多
